# Supplementary material for: Investigating the genetic contribution in febrile infection-related epilepsy syndrome and refractory status epilepticus
Source: Front Neurol. 2023 Apr 3;14:1161161. doi: 10.3389/fneur.2023.1161161 (PMC10106651; doi:10.3389/fneur.2023.1161161)
Supplement: Supplementary file 1 [file Data_Sheet_1.pdf]

Supplementary Material

The genetic spectrum of febrile infection-related epilepsy syndrome (FIRES) and refractory status epilepticus

Overview of Supplementary Material

1 Supplementary Table 1. Individuals excluded from FIRES subgroup (n=34)

2 Supplementary Table 2. Individuals excluded from FIRES subgroup with NORSE (n=7)

3 Supplementary Table 3. Genetic testing in our cohort of 25 individuals with FIRES

4 Supplementary Table 4. Individuals with RSE at initial seizure presentation

Supplementary Table 1. Individuals excluded from FIRES subgroup (n=34)

**Diagnoses for individuals excluded from the FIRES cohort**	**Individuals**	**Reason for exclusion**
Acute necrotizing encephalitis of unknown etiology	1	No fever prodrome, history of seizure 2 weeks prior
GEFS+	1	History of prior seizures
Angelman's syndrome	1	History of prior seizures
SCN2A	1	History of neonatal seizures, epilepsy
Acute necrotizing encephalitis with HHV6 virus, *RANBP2*	1	Did not have seizures
SCN1A	1	History of prior seizures
PCDH19	1	pre-existing history of seizures, prior to status presentation
Idiopathic epilepsy	4	History of prior seizures, did not have RSE
Pyruvate Carboxylase deficiency	1	Metabolic disorder
Hypoxic Ischemic Encephalopathy	2	Acquired acute symptomatic seizures
Epilepsia partialis continua	1	History of prior seizures
LIG3 mitochondrial encephalopathy	1	Did not have RSE
MOG encephalitis	1	Did not have seizures
Febrile status epilepticus	1	Fevers at onset of status epilepticus
MELAS	1	Long standing illness, prior seizures
CACNA1A	1	Febrile with seizure onset
FIRES	8	Remote history in another country lacking documentation<br>*One patient with +GAD antibodies, another with history of HSV encephalitis*
NORSE*	6	Remote history in another country/lacking documentation

* See Supplementary Table 2 for individuals excluded with NORSE.

Supplementary Table 2. Individuals excluded from FIRES subgroup with NORSE (n=7)

Individual with NORSE	Findings	Sufficient documentation for chart review
1	*PCDH19*, recent vaccination for flu ; NORSE	No; consultation without outside records
2	NORSE	No; remote history in outside country without documentation
3	NORSE	No; Telephone consult at CHOP without outside records
4	NORSE/MOG encephalitis	Yes
5	NORSE	Yes
6	NORSE	Yes

There were 3 individuals with NORSE with sufficient clinical information, therefore further analyses and clinical data on NORSE were excluded from this study.

Supplementary Table 3. Genetic testing in our cohort of 25 individuals with FIRES

Participant	Year diagnosed with FIRES (age, years)	Single Gene	Karyotype	SNP Microarray	Epilepsy Gene Panel	Exome	Mitochondrial	Other
**1**	2010<br>(7.35 years)							
**2**	2012<br>(5.06 years)							
**3**	2012<br>(7.81 years)			Normal result				
**4**	2013<br>(11.9 years)	*SCN1A* sequencing report N/A for review			• *GAMT,* c.581T>C p.(V194A), heterozygous, inheritance unknown, VOUS			
**5**	2013<br>(9.21 years)			Normal result	• *NRXN1,* c.2627C>T p.(A876V), heterozygous, inheritance unknown, VOUS<br>• *ZEB2,* c.808-4A>G p.? heterozygous, inheritance unknown, VOUS			
**6**	2013<br>(18.7 years)		Normal result	Normal result				
**7**	2013<br>(10.8 years)	*TWIST* sequencing: Negative	Normal result		Negative			
**8**	2014<br>(0.63 years)					• *SCN5A*, c.5477G>A p.(R1826H), heterozygous, inheritance unknown, pathogenic	• *HSPD1*, c.425G>A p.(A142L), heterozygous, VOUS<br>• *DARS2,* c.228-20dupT, IVS2-20dupT, heterozygous, VOUS	Maturity Onset Diabetes of the Young (MODY) Panel:<br>• *BLK,* c.26C>T p.(P9L), heterozygous, inheritance unknown, VOUS<br>WES reanalysis: no additional variants
**9**	2014<br>(6.52 years)	*SCN1A* sequencing: Negative		Normal result		• *ITPR1,* c.187A>G p.(M63V), heterozygous, de novo, VOUS<br>• *BTD*, c.1330G>C	Negative	

						p.(D444H), heterozygous, maternally inherited pathogenic<br>• *CFTR*, c.1521_1523delCTT p.(F508del), heterozygous, maternally inherited, pathogenic<br>• *COL18A1*, c.2824_2840delGGCCCCCCAGGCCCCCC, heterozygous, inheritance unknown, pathogenic<br>• *POLRIC*, c.796delG heterozygous, paternally inherited, pathogenic		
**10**	2014<br>(3.39 years)			Normal result	• *PNKP*, c.1123G>T p.(G375W), heterozygous, inheritance unknown, likely pathogenic	Duo reanalysis<br>• *CPA6*, c.919G>A p.(A307T), heterozygous, inheritance unknown, VOUS<br>• *PNKP*, c.1123G>T p.(G375W), heterozygous, inheritance unknown, pathogenic	Negative	
**11**	2014<br>(8.47 years)	*POLG* sequencing: Negative	Normal result	Normal result	N/A for review			
**12**	2016<br>(4.96 years)				• *CNTN2*, c.2735C>T p.(P912L), heterozygous, inheritance unknown, VOUS			Humoral Dysfunction Panel:<br>• *LRBA*, c.6695T>C p.(I2232T), heterozygous, inheritance unknown, VOUS
**13**	2016 (12.38 years)				Negative	N/A for review		

**14**	2016<br>(8.07 years)		Normal result		Negative	Negative	• MT-TK m.8342 G>A p.? approximately 3% heteroplasmy, likely pathogenic variant (extracted from brain tissue, approximately 4% heteroplasmy in the blood)	
**15**	2017<br>(2.39 years)	*CPT2* sequencing: Negative		Normal<br>result	Negative	• *CPA6*, c.932G>A p.(R311Q), heterozygous, maternally inherited, VOUS	Negative	
**16**	2018<br>(5.95 years)		Normal result			Negative	• *MT-ND5* m.13154T>C p.(I273T), approximately 16%, maternally inherited, VOUS	
**17**	2018<br>(2.79 years)				Negative	Negative	• *ADCK4*, c.1336G>C p.(G446R), heterozygous, inheritance unknown, VOUS<br>• *MRPS22*, c.766C>T p.(R256C), heterozygous, inheritance unknown, VOUS<br>• *TYMP*, c.1207G>A p.(G403L), heterozygous, inheritance unknown, VOUS	WES Reanalysis: negative
**18**	2018<br>(6.61 years)	*SCN1A* sequencing: N/A for review<br>*POLG* - sequencing:			• *PNPO*, c.527C>G p.(S176C), heterozygous, VOUS			

		N/A for review						
**19**	2019<br>(7.25 years)	*HLA-B*: Negative	Normal result	• 1.49Mb interstitial duplication within chromosome Xq23, inheritance unknown, VOUS		• *GRIN2D*, c.250G>C p.(V84L), heterozygous, maternally inherited, VOUS<br>• *SPTBN5*, c.2366G>A p.(R789G), heterozygous, maternally inherited, VOUS<br>• *SPTBN5*, c.749G>T p.(G250V), heterozygous, paternally inherited, VOUS		Fragile X: Negative
**20**	2019<br>(2.59 years)			Normal result	• *JMJD1C* c.3933T>C (silent mutation), het, VOUS			
**21**	2019<br>(9.84 years)					• ATP1A3, c. 719T>C p.(V240A), heterozygous, paternally inherited, VOUS	Negative	
**22**	2020<br>(17.0 years)				N/A for review	• *ADAM17*, c.1894G>A p.(V632I), heterozygous, inheritance unknown, VOUS<br>• *ADAM17*, c.178C>A p.(L60I), heterozygous, inheritance unknown, VOUS<br>• *CR2*, c.2518C>G p.(R840G), heterozygous, inheritance unknown, VOUS<br>• *NTRK1*, c.1643G>A p.(R548Q), heterozygous,	Negative	

						inheritance unknown, VOUS		
**23**	2020<br>(6.88 years)		N/A for review		Negative	• *LPA*, c.2462C>T p.(P821L), heterozygous, paternally inherited, VOUS	Negative	
**24**	2020<br>(6.34 years)				• *DIAPH1*, c.2158C>T p.(L720F), heterozygous, paternally inherited, VOUS<br>• *RANBP2*, c.1320A>T p.(L450F) heterozygous, maternally inherited, VOUS	• *FLG*, c.8911A>T p.(R2971*), heterozygous, paternally inherited, Pathogenic	Negative	
**25**	2021<br>(16.7 years)	*HBB* sequencing:<br>• *HBB*, c.92+5G>C, Intronic, heterozygous, inheritance unknown, VOUS<br>*HBA1:* Negative<br>*HBA2:* Negative	Normal result	Normal result	• *CACNA2D2*, c.227G>A p.(R76H), heterozygous, inheritance unknown, VOUS<br>• *CNTNAP2*, c.14C>T p.(P5L), heterozygous, inheritance unknown, VOUS<br>• *GTPBP3*, c.1255G>A p.(G419S), heterozygous, inheritance unknown, VOUS<br>• *PACS2*, c.2597T>C p.(V866A), heterozygous, inheritance unknown, VOUS<br>• *PEX10*, c.845T>G p.(L282W), heterozygous, inheritance unknown, VOUS<br>• *RELN*, c.576A>G (Silent), heterozygous, inheritance unknown, VOUS	• *DUOX2* c.2895_2898del p.(F966Sfs*29), heterozygous, inheritance unknown, Likely Pathogenic<br>• *SLC9A7*, c.1985T>C p.(L662P), hemizygous, maternal inheritance, VOUS		

Supplementary Table 4. Individuals with RSE identified from CHOP cohort

<table>
<tr><th>Case</th><th>Age (Sex)</th><th>Clinical presentation</th><th>Gene and variant</th><th>Outcome</th></tr>
<tr><td>1</td><td>23m (F)</td><td>Refractory focal status epilepticus in the setting of metapneumovirus</td><td>CACNA1A
p.Leu618Ser</td><td>Well-controlled epilepsy and mild developmental delay</td></tr>
<tr><td>2</td><td>4m (M)</td><td>Focal status epilepticus that was initially responsive to one dose of lorazepam, however within a couple hours his seizures recurred, requiring escalating doses of medication</td><td>CACNA1A
p.Val1396Met</td><td>Refractory epilepsy, moderate global developmental delay, autism, ataxia, and hemiplegic migraine</td></tr>
<tr><td>3</td><td>11m (F)</td><td rowspan="2">Twins born from consanguinous parents. Both presented with refractory status epilepticus in the setting of a febrile illness. Both had acute brain injury with restricted diffusion and signal abnormalities in the brainstem and deep gray regions in the brain consistent with acute necrotizing encephalitis</td><td>RANBP2
p.Asp2631Ala</td><td>Unknown</td></tr>
<tr><td>4</td><td>7m (F)</td><td>RANBP2
p.Asp2631Ala</td><td>Developmental delay</td></tr>
<tr><td>5</td><td>4m (F)</td><td>Refractory clinical SE. Received several doses of anti-seizure medications, then was found to be in subclinical status epilepticus upon initiating EEG</td><td>KCNA2
p.Pro405Leu</td><td>Neurotypical at last follow-up at 7m</td></tr>
<tr><td>6</td><td>6m (F)</td><td>RSE in the setting of fever after vaccine administration</td><td>PCDH19
p.Thr404Ile</td><td>Unknown</td></tr>
</table>
